# Scalable in-hospital decontamination of N95 filtering face-piece respirator with a peracetic acid room disinfection system

**DOI:** 10.1017/ice.2020.1257

**Published:** 2020-10-12

**Authors:** Amrita R. John, Shine Raju, Jennifer L. Cadnum, Kipum Lee, Phillip McClellan, Ozan Akkus, Sharon K. Miller, Wayne D. Jennings, Joy A. Buehler, Daniel F. Li, Sarah N. Redmond, Melissa Braskie, Claudia K. Hoyen, Curtis J. Donskey

**Affiliations:** 1 Department of Medicine, University Hospitals Cleveland Medical Center, Cleveland, Ohio; 2 Research Service, Louis Stokes Cleveland VA Medical Center, Cleveland, Ohio; 3 UH Ventures, Innovation Center, University Hospitals Cleveland Medical Center, Cleveland, Ohio; 4 Weatherhead School of Management, Case Western Reserve University, Cleveland, Ohio; 5 Department of Mechanical and Aerospace Engineering, Case Western Reserve University, Cleveland, Ohio; 6 Department of Biomedical Engineering, Case Western Reserve University, Cleveland, Ohio; 7 Department of Orthopedics, Case Western Reserve University, Cleveland, Ohio; 8 NASA Glenn Research Center, Environmental Effects and Coatings Branch, Cleveland, Ohio; 9 HX5 at NASA Glenn Research Center, Cleveland, Ohio; 10 Case Western Reserve University School of Medicine, Cleveland, Ohio; 11 Department of Environmental Health and Safety, University Hospitals Cleveland Medical Center, Cleveland, Ohio; 12 Department of Pediatric Infectious Diseases, University Hospitals Cleveland Medical Center, Cleveland, Ohio; 13 Geriatric Research, Education, and Clinical Center, Louis Stokes Cleveland VA Medical Center, Cleveland, Ohio

## Abstract

**Background:**

Critical shortages of personal protective equipment, especially N95 respirators, during the coronavirus disease 2019 (COVID-19) pandemic continues to be a source of concern. Novel methods of N95 filtering face-piece respirator decontamination that can be scaled-up for in-hospital use can help address this concern and keep healthcare workers (HCWs) safe.

**Methods:**

A multidisciplinary pragmatic study was conducted to evaluate the use of an ultrasonic room high-level disinfection system (HLDS) that generates aerosolized peracetic acid (PAA) and hydrogen peroxide for decontamination of large numbers of N95 respirators. A cycle duration that consistently achieved disinfection of N95 respirators (defined as ≥6 log_10_ reductions in bacteriophage MS2 and *Geobacillus stearothermophilus* spores inoculated onto respirators) was identified. The treated masks were assessed for changes to their hydrophobicity, material structure, strap elasticity, and filtration efficiency. PAA and hydrogen peroxide off-gassing from treated masks were also assessed.

**Results:**

The PAA room HLDS was effective for disinfection of bacteriophage MS2 and *G. stearothermophilus* spores on respirators in a 2,447 cubic-foot (69.6 cubic-meter) room with an aerosol deployment time of 16 minutes and a dwell time of 32 minutes. The total cycle time was 1 hour and 16 minutes. After 5 treatment cycles, no adverse effects were detected on filtration efficiency, structural integrity, or strap elasticity. There was no detectable off-gassing of PAA and hydrogen peroxide from the treated masks at 20 and 60 minutes after the disinfection cycle, respectively.

**Conclusion:**

The PAA room disinfection system provides a rapidly scalable solution for in-hospital decontamination of large numbers of N95 respirators during the COVID-19 pandemic.

The coronavirus disease 2019 (COVID-19) pandemic has revealed inadequacies within our healthcare systems, including the critical shortage of personal protective equipment (PPE).^1,2^ Single-use disposable PPE such as N95 filtering face-piece respirators (FFRs) and surgical face masks are being worn for extended periods or reused until they become soiled or visibly damaged. Shortages of PPE have been detrimental to the morale of healthcare workers (HCWs) and places them at risk for infection, disability, and death.^3–5^

Among all PPE, the critical shortage of N95 FFRs has been most pronounced.^6,7^ At the onset of the outbreak, the Centers for Disease Control and Prevention (CDC) recommended that N95 FFRs be used for all interactions with confirmed or suspected COVID-19 patients. The CDC subsequently modified its guidance regarding PPE required while caring for patients with COVID-19.^8^ Presently, both the CDC and the World Health Organization (WHO) recommend the use of N95 FFRs for all aerosol-generating procedures (AGPs) performed on confirmed COVID-19 patients and persons under investigation (PUI).^9,10^ Given the shortage of N95 respirators, the CDC has provided updated guidance for extended use and limited reuse of these respirators by HCWs.^11^ Several strategies have been proposed for conserving PPE: repurposing other devices to be used as FFRs; creating FFRs at home; and decontaminating N95s using ultraviolet-C germicidal irradiation, dry heat, moist heat, or vaporized hydrogen peroxide.^12–15^ Vaporized hydrogen peroxide (VHP) was given provisional US Food and Drug Administration (FDA) emergency use authorization (EUA) for the decontamination of used N95 respirators.^16^ However, VHP decontamination is a labor- and time-intensive process due to a long treatment cycle, and it requires the shipment of used N95 respirators to a central-processing center.^17^ The FDA has also granted an EUA for other sterilization devices that are currently in use in several hospitals. This EUA allows for in-hospital disinfection of used N95 FFRs; however, these devices are limited by the number of N95 FFRs that can be processed at a given time.

An effective N95 respirator disinfection process that will allow on-site reprocessing with rapid turnaround times, ease of use with existing personnel expertise, and scalability to process large quantities of respirators is urgently needed. We previously reported that a high-level disinfection cabinet that generates aerosolized peracetic acid (PAA, also known as peroxyacetic acid) and hydrogen peroxide was effective for disinfection of N95 respirators.^18,19^ Here, we expanded on these promising findings by evaluating the use of this technology on a larger scale.

## Methods

A multi-institutional study was conducted at University Hospitals Cleveland Medical Center (UHCMC), Case Western Reserve University (CWRU), National Aeronautical and Space Administration (NASA) Glenn Research Center, and the Cleveland Veterans’ Affairs Medical Center (VAMC) to evaluate the use of an ultrasonic room disinfection system that generates aerosolized PAA and hydrogen peroxide for disinfecting large numbers of N95 respirators.

### Protection of human research participants

The proposed PAA disinfection experiments were approved by an internal safety review at University Hospitals Cleveland Medical Center (UHCMC). The microbiologic analyses were approved by the Biosafety Committee at the VAMC. Institutional review board approval was not obtained because human subjects were not enrolled in the study.

### Development and optimization of the PPE decontamination room

**Fig. 1.**
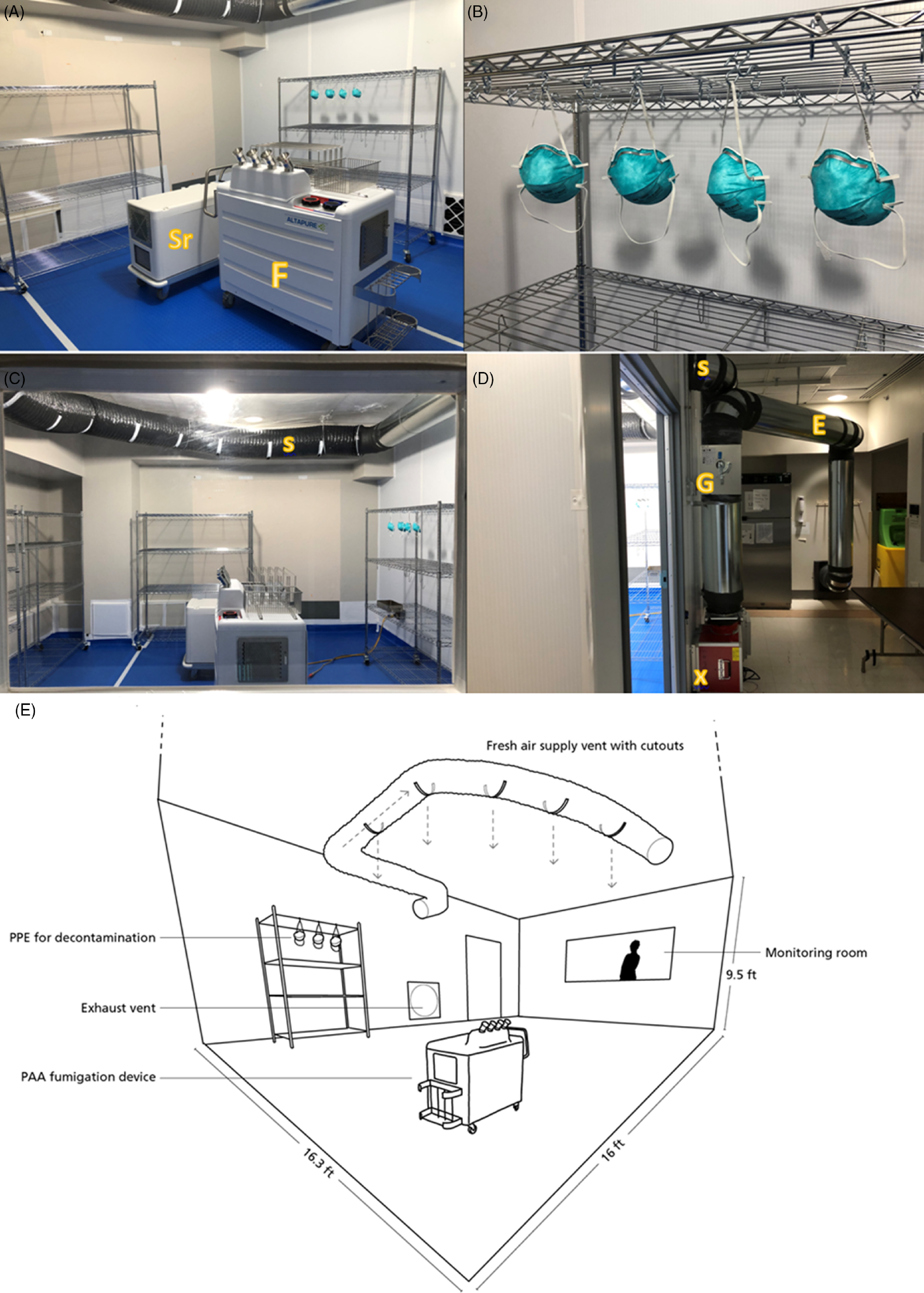
(A) Placement of the aerosolization device (F) and extra scrubber (Sr) in the middle of the test room. (B) N95 masks suspended on ‘S’ shaped hooks. (C) Test room layout with ventilation on the ceiling providing fresh air (supply) into the room during the vent cycle. (D) The ventilation set up with supply (S) and exhaust (E). Note two 600-cfm blower fans (X) in a push–pull configuration with manually operated gasketed dampers (G). (E) Schematic diagram of the room dimensions and ventilation system.

The PAA high-level disinfection system (HLDS; AP-4, Altapure, Mequon, WI) was placed in the center of a room measuring 16.3 feet × 16 feet × 9.5 feet (2,447 cubic feet; 5 m × 4.8 m × 2.9 m; 69.6 cubic meters) (Fig. 1A–E). The device uses ultrasonic vibrations to generate a dense cloud of submicron droplets of PAA, consisting of peracetic acid (0.18%), hydrogen peroxide (0.88%), water (98.58%), and the remainder is inert ingredients.^20^ The aerosol eventually decomposes into nontoxic end products: water vapor, acetic acid (vinegar), and oxygen. The decontamination cycles consisted of 4 phases: an aerosol deployment phase (ie, release of PAA submicron aerosols into the room), a dwell phase (ie, aerosols left to stand in the room), a scrub phase (ie, aerosol is dehumidified and drawn through activated charcoal filters), and a vent phase (ie, fresh air is allowed to circulate by opening the manual vents enabling clearance of residual vapors and drying of the masks). The ventilation in the test room was modified to allow the influx and circulation of fresh air at the end of the scrub cycle. An extra air scrubber (HJ-200, Altapure, Mequon, WI) was deployed to minimize vent times by accelerating the clearance of residual PAA. The deployment and dwell times are directly responsible for microbial reduction, whereas the scrub and the vent cycles influence the clearance of residual PAA vapors to recommended safety levels. There are no specific OSHA standards for PAA.^21^ The American Conference of Governmental Hygienists (ACGIH) has set a threshold limit value (TLV) of 0.4 ppm as a 15-minute short-term exposure limit (STEL).^21–23^ The acute exposure guideline (AEGL-1) limit recommended by the US Environmental Protection Agency (EPA) is 0.17 ppm (0.52 mg/m^2^).^24^

Before the start of the disinfection cycle, the aerosol deployment, dwell, scrub, and vent times were manually configured using the application programming interface (API). The deployment and dwell times were adjusted to provide effective disinfection of the masks with the least amount of exposure to PAA. The PAA concentrations in the room were measured in real time using a PAA sensor (Safecide, ChemDAQ, Pittsburgh, PA). At the end of the vent cycle, the PAA concentrations were 0.12 ppm, below the AEGL-1 limit of 0.17 ppm. The 15-minute time-weighted average of PAA concentration after the decontamination cycle was 0.08 ppm, well below the 15-minute STEL of 0.4 ppm.

We evaluated 3 test cycles to identify a cycle time that achieved consistent disinfection of bacteriophage MS2 and *G. stearothermophilus* spores inoculated onto N95 respirators. The shortest cycle tested was 44 minutes: deployment phase, 12 minutes; dwell phase, 8 minutes; scrub phase, 8 minutes; and vent phase, 16 minutes. Based on these results, the cycle times were incrementally adjusted to achieve an optimal cycle time. The optimal cycle was identified as the shortest cycle at which disinfection was consistently achieved: deployment phase, 16 minutes; dwell phase, 32 minutes; scrub phase, 12 minutes; and vent phase, 16 minutes. This cycle was then repeated up to 5 times with sterile masks that were subsequently analyzed for structural integrity and instantaneous filtration efficiency and underwent load testing.

### Efficacy of the decontamination process for treatment of contaminated N95 respirators

The model 1860 N95 (3M, Minneapolis, MN) respirator was studied because it was the respirator used at the study hospital. In total, 20 new respirators were tested from 2 different lots from the hospital inventory. Two N95 respirators were used for each decontamination test cycle. The test and control respirators were inoculated with ~10^6^ colony-forming units (CFU) of *G. stearothermophilus* spores and ~10^6^ plaque-forming units (PFU) of bacteriophage MS2 on the outer and inner surfaces of the respirator as previously described.^18,25,26^ The test organisms were suspended in 8% simulated mucus,^27^ and 10 µL aliquots were pipetted onto the respirator surface and spread with a sterile loop to cover an area of 1 cm^2^ and allowed to air dry. The test N95 respirators were suspended using metallic ‘S’ shaped hooks from shelving carts at a height of ~2 m and exposed to PAA submicron droplets. The control masks were left untreated at room temperature, maintained at 20°C (68°F).

Following disinfection treatments, the inoculated sections of the N95 respirators were cut out and vortexed for 1 minute in 1 mL phosphate-buffered saline with 0.02% Tween 80. Serial dilutions were then plated on selective media to quantify viable organisms. Broth enrichment cultures were used to assess for recovery of low levels of *G. stearothermophilus* spores. All tests were performed in triplicate. Log_10_ CFU or PFU reductions were calculated by comparing recovery from treated versus untreated control respirators.

### Evaluation of contact angle on the surface of treated N95 respirators

The contact angle on the surface of untreated and treated N95 respirators was measured with a contact angle meter (Kernco Instruments, El Paso, TX). A micropipette was used to place a small droplet of deionized water on the surface (outer green layer) of a ~1.2-cm × 2.5-cm section cut from each mask with scissors. Contact angle (θ) for each of 3 drops was measured using the goniometer scale on the instrument for each sample and the range of angles documented.

### Evaluation of N95 respirator structural integrity by scanning electron microscopy

The outer (green) fabric of the mask was examined using scanning electron microscopy (SEM). Samples of ~2.5 cm × 2.5 cm cut from each mask with scissors were coated with a 10-nm layer of platinum to reduce charging in the electron beam and then mounted to a ~10-cm pin-mount platen with conductive carbon tape for SEM viewing. A Tescan MAIA-3 Scanning Electron Microscope was used to view the fibers in each mask sample. The test parameters were set as follows: accelerating voltage, 1 kV; working distance, 15 mm; beam intensity, 8 (resulting in an absorbed current of ~180 pA); and spot size, ~28 nm.

### Effect of treatment on elasticity of the respirator straps

At each sterilization cycle, 3 samples (3 cm long) were cut from elastic straps of 2 masks (n = 6 per group). Samples were clamped at a materials testing machine (Testresources, Minnetonka, MN) and were loaded for 2 consecutive loading and unloading cycles under tension at a rate of 1 mm/s. The testing profile included 2 consecutive cycles of load relaxation such that the sample was stretched 3 times the original length, was held at constant deformation for 5 minutes, and was unloaded. Load values at peak deformation (‘load 1’ and ‘load 2’) and load-relaxation values for each cycle (‘relaxation 1’ and ‘relaxation 2’) were recorded. As such, relaxation represents the capacity of straps to retain a load over time. The elasticity of samples was from the slope of the line connecting the zero load with the peak load in the load deformation plot. A nonparametric Kruskal-Wallis test was used at a significance level of *P* < .05.

### Filtration efficiency of the N95 masks following exposure to PAA vapor

Evaluation of filtration efficiency was performed at ICS Laboratories (Brunswick, OH). N95 respirators subjected to multiple runs of the optimal cycle were subjected to testing for filtration efficacy in accordance with NIOSH standard TEB-APR-STP-0059.^28^ The masks were conditioned for 25 hours and were then subjected to instantaneous aerosol loading. Upon exhibiting instantaneous filtration efficiency exceeding 95%, the remaining respirators were subjected to full loading. Flow rate, initial resistance, and initial penetration data were recorded.

### Measurement of PAA and hydrogen peroxide off-gassing after disinfection

**Fig. 2.**
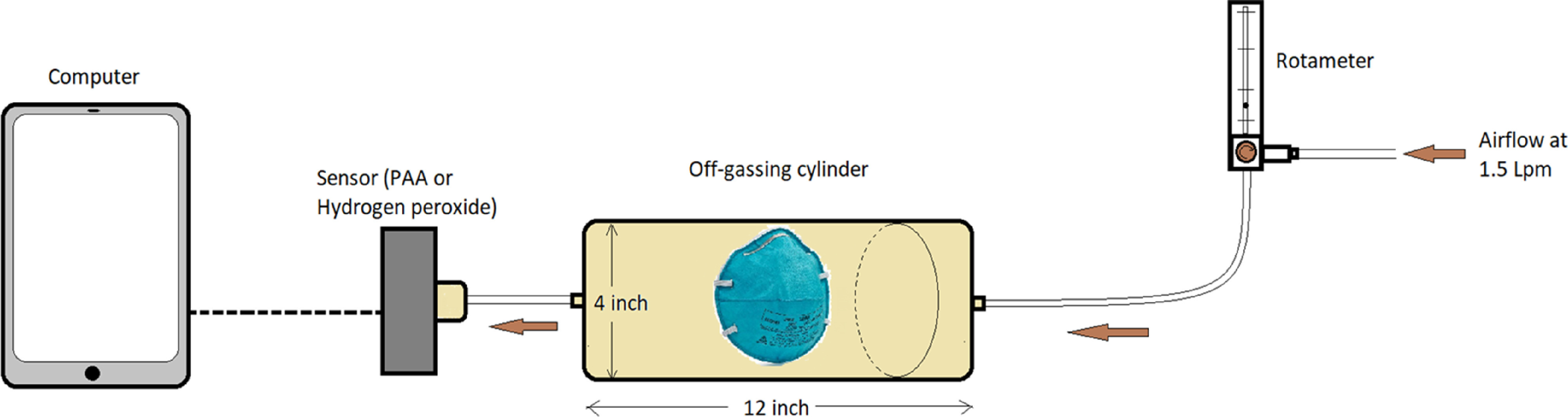
The off-gassing set up. The N95 FFR was placed in a sealed polyvinyl chloride cylinder (0.35 cu. ft.) with airflow at 1.5 L/minute entering through one end and a peracetic acid or hydrogen peroxide sensor (Safecide, ChemDAQ, Pittsburgh, PA) connected to the other end. A 15-minute time-weighted average (TWA) for peracetic acid or hydrogen peroxide exposure was measured.

Following the optimal disinfection cycle, an N95 FFR was taken out of the decontamination room and allowed to air dry in a room with a fan blowing at 500 cubic feet per minute (cfm; 1.41 cubic meters per minute). The N95 FFRs were tested for off-gassing after the optimal disinfection cycle and at 20-minute intervals. Testing was concluded once 2 consecutive tests showed no off-gassing for PAA or hydrogen peroxide. Figure 2 depicts the off-gassing set up.

## Results

### Efficacy of PAA disinfection of N95 masks

**Fig. 3.**
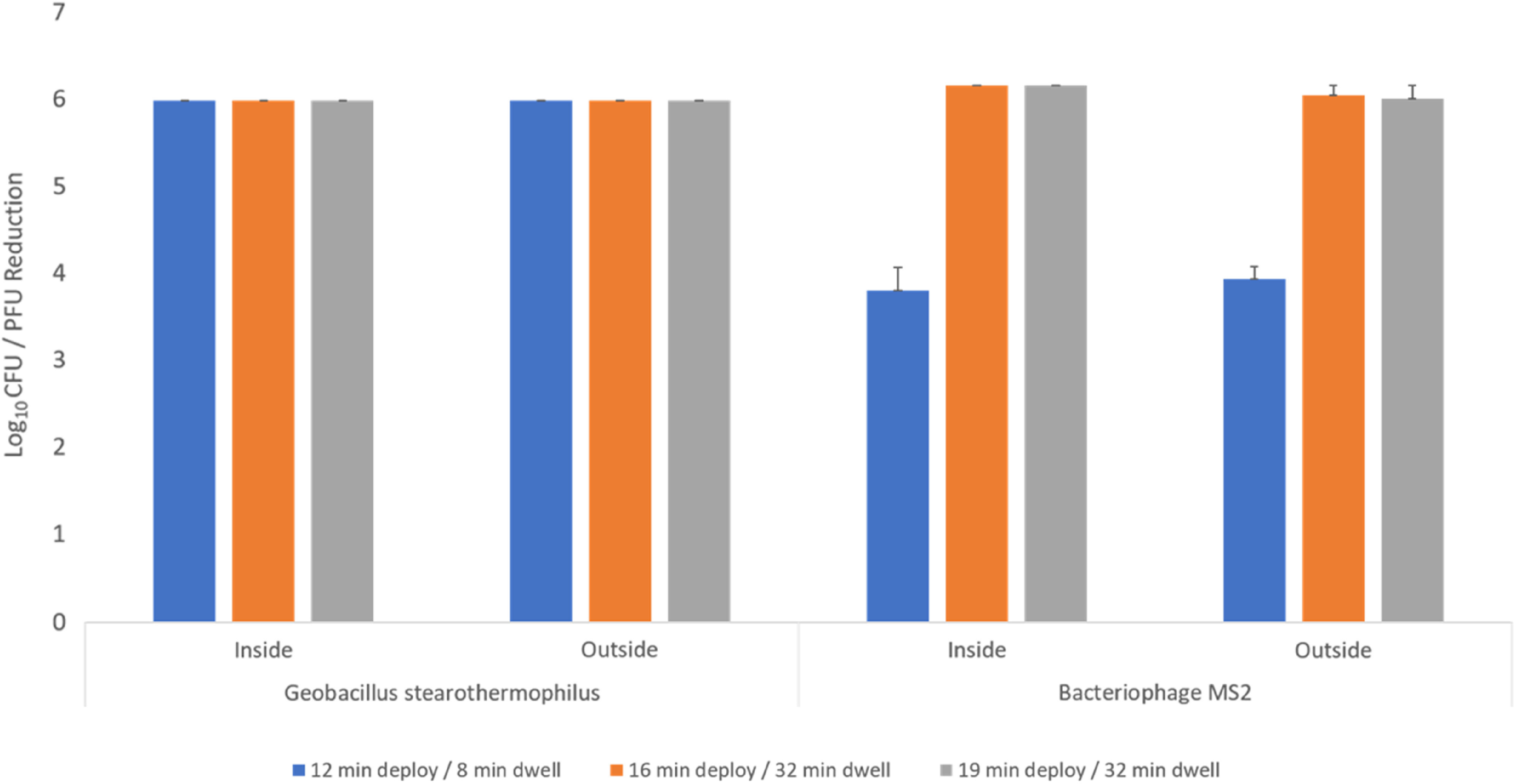
Efficacy of PAA HLDS for decontamination or disinfection of *Geobacillus stearothermophilus* spores and MS2. The respirator was exposed to 3 different cycles as in the figure and log10 reductions CFU/PFU studied. Error bars indicate standard error.

As shown in Figure 3, 6 log_10_ reductions in *G. stearothermophilus* spores were achieved on inoculated 1860 N95 FFRs with all cycle durations. For bacteriophage MS2, 6 log_10_ reductions were achieved on the inoculated 1860 N95 FFRs with the 16-minute aerosol deployment phase and the 32-minute dwell phase (total cycle time, 76 minutes) and the 19-minute aerosol deployment phase and 32-minute dwell phase (total cycle time, 87 minutes). Based on these results, the optimal disinfection cycle time was determined to be a deployment time of 16 minutes and a dwell time of 32 minutes.

### Structural integrity of the N95 masks following exposure to aerosolized PAA

**Fig. 4.**
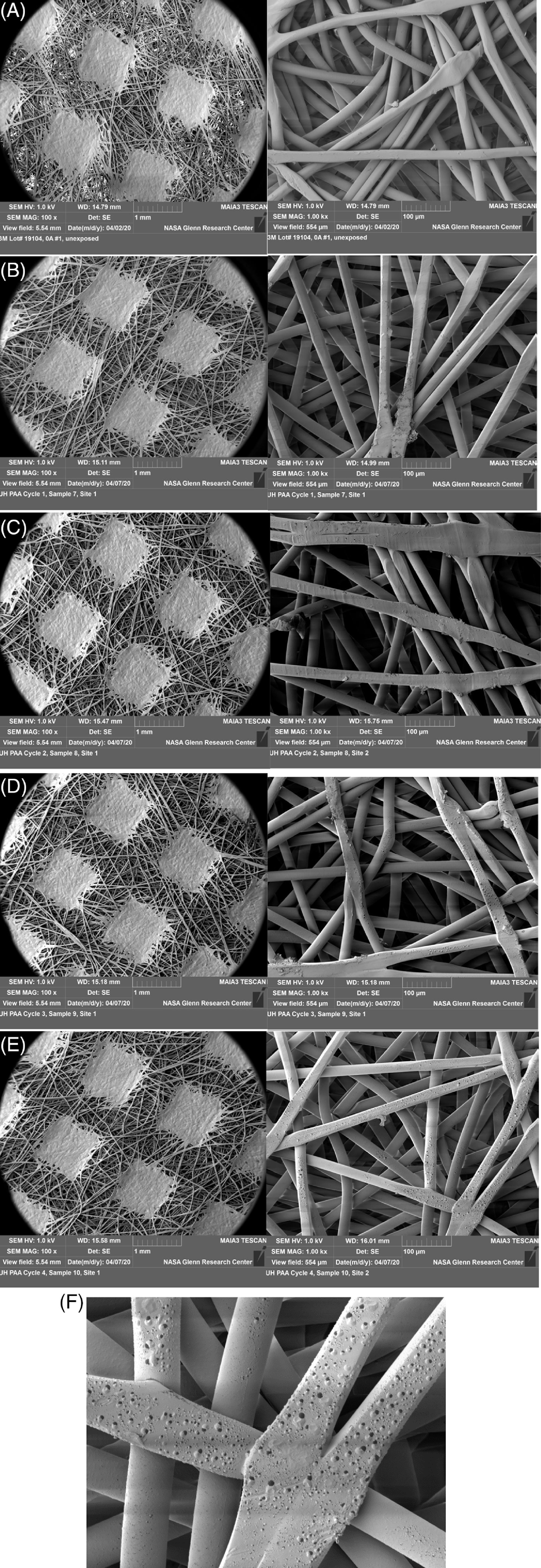
Scanning electron microscope (SEM) images of the outer layer of the N95 mask under 100× (images in the left column) and 1,000× magnification (images in the right column. (A) Control. (B–E) Multiple cycles of PAA treatment from 1 to 4. Note increase bubbling on the fibers after PAA exposure. (F) Magnified image of bubbling on fibers.

An SEM analysis revealed evidence of bubbles on the surface of the PAA-treated respirator outer fabric fibers, which appeared to increase with the number of PAA cycles (Fig. 4A–F). Energy dispersive spectroscopy dot map images of the bubble feature on PAA cycle 4 outer mask fabric indicated that the bubbles were high in oxygen, phosphorous, and nitrogen, based on the bright areas of the dot map images. The overall spectrograph showed that the surface was predominantly carbon, oxygen, and phosphorous.

**Fig. 5.**
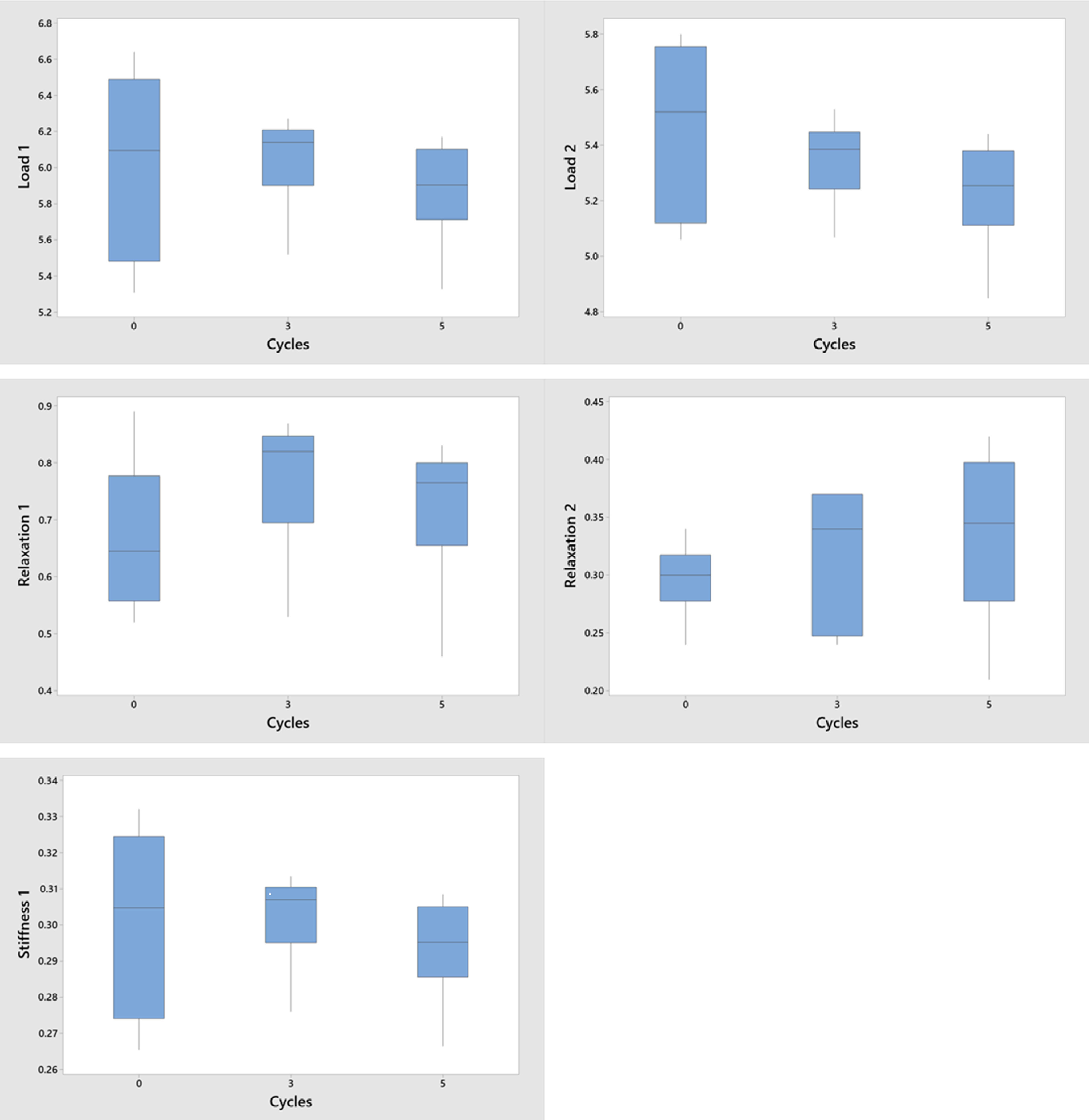
Interval plots for mechanical test variables as a function of disinfection cycles. The horizontal line is the median. The box indicates the interquartile range. The whiskers extend to the minimum and maximum values.

### Evaluation of the contact angle on the surface of the treated N95 masks

The contact angle remained at 97–99° with repeated cycles of PAA disinfection. We thereby concluded that the hydrophobicity of the outer layer was preserved.

### Effect of treatment on elasticity of the respirator straps

Elasticity of straps (as reflected by stiffness) and the capacity of straps to retain load over time (as reflected by relaxation) were not affected by the number of sterilization cycles (Fig. 5). *P* values ranged from 0.27 to 0.505.

### Filtration efficacy of the N95 masks following exposure to PAA vapor

Table 1A, 1B shows the results of filtration efficiency on the masks subjected to 5 cycles of PAA treatment. We did not detect a decrease in filtration efficiency for up to 5 cycles of PAA disinfection.

**Table 1A.** The Results of the Instantaneous Loading Tests for Filtration Efficiency^a^

No. of Cycles Repeated	Flow Rate, L/min	Initial Resistance, mm H_2_O	Instantaneous Penetration, %	Instantaneous Efficiency, %	Result
3×	86	12.9	0.59	99.41	Pass
3×	86	13.5	0.31	99.69	Pass
5×	86	14	0.51	99.49	Pass
5×	86	13.3	0.84	99.16	Pass
Specification	81–89			≥ 95	

a The cycle length was a dwell of 16 minutes and a deploy of 32 minutes (optimal cycle). ‘N’× indicates number of times the N95 FFR was treated with this cycle.

**Table 1B.** The Results of the Full Loading Tests for Filtration Efficiency^a^

No. of Cycles Repeated	Flow Rate, L/min	Initial Resistance, mm H_2_O	Initial Penetration, %	Maximum Penetration, %	Filter Efficiency, %	Result
5×	85	14.9	0.56	1.35	98.65	Pass
5×	86	13.8	0.6	1.37	98.63	Pass
Specification	81–89			≤ 5.0	≥ 95	

a The cycle length was a dwell of 16 minutes and a deploy of 32 minutes. ‘N’× indicates the number of times the N95 FFR was treated with this cycle.

### Results of PAA and hydrogen peroxide off-gassing after disinfection

At 20 minutes after the optimal disinfection cycle, the PAA off-gassing was measured at 0.00 ppm. At 60 minutes after the optimal disinfection cycle, the hydrogen peroxide off-gassing was measured at 0.00 ppm. Table 2 lists the full results.

**Table 2.** The Results of the Hydrogen Peroxide Off-Gassing From the N95 FFR After an Optimal Disinfection Cycle

Time After Cycle	Instantaneous	15-Min STEL
0	2.4 ppm	1.76
20 min	1.4 ppm	1.15
40 min	0.2 ppm	0.18 ppm
60 min	0.0 ppm	0.0 ppm
80 min	0.0 ppm	0.0 ppm

Note. FFR, filstering face-piece respirator; STEL, short-term exposure limit.

## Discussion

The goal of this investigation was to address the urgent need for an effective N95 respirator decontamination process allowing onsite reprocessing with rapid turnaround times, ease of use, and scalability to process large numbers of respirators. We found that the PAA room disinfection system was easy to set up and operate and that it was effective for disinfection of N95 respirators with a total cycle time of 1 hour and 16 minutes.

Microbiological agents chosen to test for disinfection were based on guidance provided by the FDA EUA document.^29^ During the Ebola outbreak, the CDC recommended the use of disinfectants that were registered to be effective against nonenveloped viruses (compared to enveloped viruses) such as SARS-CoV-2 because they were more resistant to disinfection.^30,31^ Bacteriophage MS2 is a nonenveloped virus that has been used as a surrogate in studies investigating airborne RNA viral pathogens as well as disinfectant studies performed by the US EPA.^32–34^ *Geobacillus stearothermophilus* spores have been used in the study of PAA decontamination of surfaces.^35^

Our findings are consistent with previous studies that have demonstrated the efficacy of the PAA disinfection system. Both the room HLDS and a high-level disinfection cabinet were effective in reducing pathogens, including *C. difficile* spores, on steel-disk carriers by >6 log_10_ CFU.^25,36^ However, an extended cycle with the disinfection cabinet was required to achieve a 6 log_10_ reduction in bacteriophage MS2 inoculated on N95 respirators.^18^ In the current study, an extended cycle (identified as the optimal disinfection cycle in our experiments) was also required to achieve a 6 log_10_ reduction in bacteriophage MS2 or *G. stearothermophilus* spores on N95 respirators. Similarly, Battelle^17^ reported a prolonged VHP cycle time with a total time of 480 minutes for N95 decontamination.

Our results demonstrate that the N95 respirators retain their structural integrity, outer surface hydrophobicity, and strap elasticity for at least 5 repeated cycles of PAA treatment. However, on a microscopic level, we observed evidence of visible bubbling on the nonwoven polypropylene fibers of the outer layer, which increased proportionally with the duration of exposure to PAA. The significance of these bubbles is unclear at this time. It could be indicative of a trend toward loss of structural integrity with continued exposure to PAA. These changes, however, did not affect the filtration efficiency of the treated masks. Off-gassing of PAA from the treated mask was undetectable after just 20 minutes of air drying. This finding may be explained by the inherently unstable nature of the compound leading to its rapid decomposition.^17,37^ Hydrogen peroxide off-gassing was undetectable after 60 minutes of air drying.

The PAA room disinfection system offers several advantages over other technologies being evaluated for PPE decontamination. The technology is substantially more effective than ultraviolet-C (UVC) light for N95 decontamination.^18,38^ The aerosols allow complete coverage of all surfaces on the masks, thus eliminating the concerns about ‘shadow areas’ with UVC germicidal irradiation.^39^ Compared to VHP, the cycle times with PAA are shorter with rapid turnaround times.^17,40^ This time savings can be vital for healthcare systems to achieve decontamination of large numbers of N95 FFRs. The platform is scalable and can be replicated in real-world hospital settings. We conservatively estimate that ~2,000 N95 respirators can be effectively disinfected in a room with the dimensions of the test room (2,447 cubic feet or 69.6 cubic meters), with capacity increasing in proportion to the room dimensions. An estimated 15,000–20,000 N95 FFRs can be decontaminated per day with this method. The disinfection room can be set up relatively easily with simple modifications to the ventilation setups in most hospital rooms. The device can be operated with minimal training. The PAA room HLDS is currently used for terminal disinfection of patient rooms in some centers across the United States and abroad and can be readily repurposed for N95 decontamination without much added cost.

The PAA room disinfection system has some disadvantages. The PAA aerosols are hazardous: the ventilation system must be closed and the room must be sealed during operation. The AP-4 HLDS is designed to disinfect rooms of varying sizes, but a single device does not effectively disinfect spaces >4,000 cubic feet (113.25 cubic meters). However, the software allows for synchronous use of multiple AP-4 devices if larger decontamination room setups are considered.

Our study has some limitations. Only 1 model of N95 FFR was evaluated. Construction and materials of N95 respirators vary; thus, further studies are needed with other models. Our sample size was small, in keeping with the need to preserve N95 FFRs for HCWs. We only evaluated N95 structural integrity and filtration efficiency for up to 5 treatment cycles. Despite these limitations, our study has the advantage of including assessments by a multidisciplinary group which helped evaluate the different factors that would affect the reusability of an N95 FFR.

In conclusion, we found that a PAA room HLDS was effective for the decontamination of N95 respirators with a short cycle time. No adverse effects on filtration efficiency, structural integrity, or strap elasticity were detected after 5 treatment cycles. The PAA room HLDS system provides a rapidly scalable solution for hospitals requiring in-hospital disinfection of N95 respirators.
